# Improving adenoviral vectors and strategies for prostate cancer gene therapy

**DOI:** 10.6061/clinics/2018/e476s

**Published:** 2018-08-03

**Authors:** Rodrigo Esaki Tamura, Igor Vieira de Luna, Marlous Gomes Lana, Bryan E Strauss

**Affiliations:** Laboratório de Vetores Virais, Centro de Investigação Translacional em Oncologia, Instituto do Cancer do Estado de Sao Paulo (ICESP), Hospital das Clinicas HCFMUSP, Faculdade de Medicina, Universidade de Sao Paulo, Sao Paulo, SP, BR

**Keywords:** Prostate Cancer, Adenovirus, p53, Chemotherapy, Gene Therapy

## Abstract

Gene therapy has been evaluated for the treatment of prostate cancer and includes the application of adenoviral vectors encoding a suicide gene or oncolytic adenoviruses that may be armed with a functional transgene. In parallel, versions of adenoviral vector expressing the p53 gene (Ad-p53) have been tested as treatments for head and neck squamous cell carcinoma and non-small cell lung cancer. Although Ad-p53 gene therapy has yielded some interesting results when applied to prostate cancer, it has not been widely explored, perhaps due to current limitations of the approach. To achieve better functionality, improvements in the gene transfer system and the therapeutic regimen may be required. We have developed adenoviral vectors whose transgene expression is controlled by a p53-responsive promoter, which creates a positive feedback mechanism when used to drive the expression of p53. Together with improvements that permit efficient transduction, this new approach was more effective than the use of traditional versions of Ad-p53 in killing prostate cancer cell lines and inhibiting tumor progression. Even so, gene therapy is not expected to replace traditional chemotherapy but should complement the standard of care. In fact, chemotherapy has been shown to assist in viral transduction and transgene expression. The cooperation between gene therapy and chemotherapy is expected to effectively kill tumor cells while permitting the use of reduced chemotherapy drug concentrations and, thus, lowering side effects. Therefore, the combination of gene therapy and chemotherapy may prove essential for the success of both approaches.

## Prostate cancer

In the United States, prostate carcinoma is the most frequent cancer among men, accounting for 19% of cancers in 2017. Though incidence rates vary by region and are higher in developed countries, an estimated 1.1 million men were diagnosed with prostate cancer in 2012, making it the second most common cancer in men worldwide. The highest rates are in Australia, North America, and Northern and Western Europe, which are regions where testing for prostate-specific antigen (PSA) has become commonplace. In South America, the rate is 60.1 per 100,000 (age-standardized rate). In a global estimate, prostate cancer is the fifth leading cause of death from cancer in men. The prediction for 2020 is 1,392,727 new cases worldwide 1,2.

If detected early while locally confined, prostate cancer is largely curable by radical prostatectomy or radiotherapy 3. However, initial diagnosis of up to 15% of patients include metastatic lesions, and recurrence after conventional radical therapy occurs in up to 40% of patients 4,5. Typically, androgen deprivation therapy (ADT) is given to patients with recurrent disease and is often effective, though most of these patients will relapse after 2-3 years due to the development of castration-resistant prostate cancer (CRPC) 6. The most important driver of resistance is the androgen receptor (AR), whose hyperactivity may arise through multiple mechanisms, such as AR amplification and hypersensitivity, AR mutation leading to promiscuity, androgen-independent AR activation and intratumoral alternative androgen production 7. Docetaxel, a cytotoxic antimicrotubule agent that binds to the β-tubulin subunit of microtubulin, is the first line treatment for CRPC. Although docetaxel may be effective, only approximately 48% of patients responded to combined docetaxel and prednisone treatment, with a median survival of 2.5 months over the control group 8. Other current chemotherapies include abiraterone and enzalutamide (second-generation antiandrogens), which are used as second-line treatments for CRPC, and cabazitaxel (second-generation tubulin-binding taxane) has proven to be beneficial even as a third-line treatment 9,10. Even with these advances, the 5-year survival rate of CRPC is only 31% 11; therefore, continued study of alternative therapies is warranted.

## Gene therapy for prostate cancer

Gene therapy using adenoviral vectors has been evaluated in 17 clinical trials for the treatment of prostate cancer (clinicaltrials.gov). Here, we will address some of these approaches, emphasizing the modifications made to the vector. The use of a prostate-specific promoter to drive viral expression or control oncolytic viral replication assures specificity through transcriptional control. Alternatively, modifications in the fiber protein are expected to alter vector tropism at the level of transduction.

Most of the prostate-specific promoters are derived from the PSA and prostate-specific membrane antigen (PSMA) enhancers 12,13. A chimera composed of the PSA enhancer and the probasin (PB) promoter showed 20% less activity than the cytomegalovirus (CMV) promoter but high prostate cancer cell specificity 14. Promoters of tyrosinase 15, human glandular kallikrein (hKLK2) 16, PB and the mouse mammary tumor virus long terminal repeat (MMTV LTR) 17 have also been tested for their usefulness in treating prostate cancer.

The serotype 5 adenovirus (Ad5) initially makes contact with its cellular receptor (coxsackievirus and adenovirus receptor, CAR) through the fiber protein; thus, its modification can direct viral tropism. For example, compared to an unmodified Ad5 vector, a shortened chimeric Ad5/35 hybrid fiber protein had an increased transgene insert size as well as increased transduction efficiency in different prostate cancer cell lines 18. A fiber knob chimera of Ad5/3 facilitated transduction in a CAR-independent manner 19. Incorporation of the Arg-Gly-Asp (RGD) tripeptide motif into the fiber protein increased transduction, even in CAR-negative prostate cancer cell lines 20,21. Additionally, tissue-specific expression was enhanced in high-capacity adenoviral vectors compared to first-generation adenoviral vectors 15. To avoid destruction of the virus particles by the immune system, use of other adenovirus serotypes (Ad6), shielding of the virus particle with PEG and use of mesenchymal stem cells (MSCs) and dendritic cells as carriers have been tested 22-24.

Conditionally replicating viruses were tested in phase I/II clinical trials. These adenoviral vectors encode the E1 gene under control of a tissue-specific promoter, thus limiting viral replication. In a phase I clinical trial with 20 patients, different doses of the CG7060 virus (Cell Genesys, South San Francisco, CA), which contains the PSA enhancer, were well tolerated, and there was a correlation between viral dose and effect: the patients with the greatest reduction in PSA levels received the highest dose 25. Another vector, CG7870 (Cell Genesys), has the E1 gene under control of the rat PB promoter and the E1B gene under control of the PSA promoter, providing two degrees of specificity. The CG7870 vector was administered in 23 patients with CRPC, and 5 patients showed a 25-49% decrease in PSA levels 26. The combination of CG7870 with taxanes resulted in a synergistic response *in vitro* 27. Another promoter used is a chimera composed of the PSA and PSMA enhancers, as well as the T-cell receptor γ-chain alternate reading frame protein promoter, Ad[I/PPT-E1A]. While not yet tested clinically, the Ad[I/PPT-E1A] oncolytic vector showed prostate-specific activity in both a hormone-dependent and hormone-independent manner, suggesting its usefulness even in patients treated with androgen withdrawal 28. A vaccination protocol using an adenoviral vector expressing PSA was used in a phase I clinical trial and was shown to be safe and to increase survival in CRPC patients 29. Phase II clinical trials are underway 30.

Oncolytic adenoviral vectors have also been armed with functional transgenes. A prostate-restricted replicative adenovirus (PRRA) using a prostate-specific enhancer (PSES) to control the expression of E1A, E1B and E4 was armed with the FasL gene. This conditionally replicating adenoviral vector induced apoptosis of PSA/PMSA-positive cells and was less toxic compared to an AdCMVFasL vector, which killed all mice in 16 hours due to multivisceral failure 31. PRRAs have been armed with other transgenes such as a fusion of the endostatin and angiostatin genes 18, reduced expression in immortalized cells (REIC) 32, glioma pathogenesis-related protein 1 (GLIPR1) 33 and a fusion protein of PSA and CD40L 34.

First-generation adenoviral vectors armed with a suicide gene were safe and well tolerated in phase I clinical trials for patients with local recurrent or metastatic prostate cancer. Only one patient who received the highest viral dose suffered grade 4 thrombocytopenia and grade 3 hepatotoxicity; some patients showed evidence of reduced PSA levels. Suicide gene therapy has also been combined with conditionally replicating adenoviruses in prostate cancer patients with locally recurrent disease, newly diagnosed and locally aggressive disease or metastatic disease. Even though it was shown to be safe and an initial PSA decline was observed, patients relapsed 35-39. Long-term follow up of the patients with local recurrence who were treated with a combination of suicide gene therapy and radiotherapy showed that the patients benefited in terms of the PSA doubling time (PSADT) 40. Introducing suicide therapy into a second-generation adenovirus in combination with intensity-modulated radiotherapy (IMRT) in patients with newly diagnosed prostate cancer resulted in low toxicity and a reduction in the percentage of patients who were positive for adenocarcinoma 41. Ultrasound-directed intraprostatic injection of an adenoviral vector expressing thymidine kinase (Ad-TK) was well tolerated and none of the 10 patients with adenocarcinoma developed metastases 42. Strategies involving suicide gene therapy in prostate cancer are ongoing (clinicaltrials.gov).

## ADENOVIRUS-P53 IN PROSTATE CANCER

### P53 in prostate cancer

The p53 protein is an important tumor suppressor involved in a variety of cellular responses to stress. P53 degradation is mediated by murine double minute 2 (MDM2), and disruption of the p53/MDM2 complex frees p53 to promote the transcription of specific target genes that, in turn, direct cellular responses such as apoptosis 43,44. In prostate cancer, p53 alterations occur in approximately 5% of cases, while this number rises to 65% in metastatic disease 45,46. The detection of p53 in prostate cancer was analyzed in more than 50 studies showing that immunohistochemical staining of p53 increases in high-grade carcinomas, advanced stage cancer and carcinomas of peripheral zone origin 47. However, there are fewer studies mapping the alterations in p53 in prostate cancer, and such studies show divergent frequencies ranging from 3-40% in p53 gene mutation and 10-60% deletions or loss of heterozygosity 48-50. A more recent study that combines tissue microarray (TMA) and DNA analysis found that different types of p53 alterations characterize subgroups of prostate cancer with distinctively different prognoses; strong p53 immunostaining is rare but represents an independent and worse prognostic event in prostate cancer 47.

### Ad-p53 clinical trials

Viral vectors expressing p53 have been in development for the last 25 years, and even though retroviral vectors were the first to be tested in patients, adenoviral vectors have been more broadly used, and different versions have been employed to express the tumor suppressor p53 51-54. Three slightly different first-generation adenoviral vectors reached clinical trials; all with deletions in the E1 gene and expression of the p53 gene under control of constitutive promoters (CMV or Rous sarcoma virus [RSV]). These Ad-p53 vectors known as Advexin (Introgen Therapeutics, Multivir, Inc, both of Houston, TX), SCH58500 (Merck & Co; Schering-Plough, Kenilworth, NJ) and Gendicine (Shenzhen SiBiono GeneTech, Guangdong, China) have been tested for treating different types of cancers, including non-small cell lung cancer (NSCLC), head and neck squamous cell carcinoma (HNSCC), colorectal, bladder and several other cancers 51,. Even with the publication of promising clinical results, only Gendicine has been approved for commercialization and is currently being used in China for the treatment of head and neck cancer. A phase I clinical trial using Advexin for prostate cancer showed that the vector is safe, with no grade 3 or 4 side effects, and that the vector induced the expression of p53 and apoptosis of the tumor cells 64. Even so, no further trials testing Advexin for the treatment of prostate cancer were performed.

### Improvement of Ad-p53 for prostate cancer

In the 1990s, Ad-p53 was tested in prostate carcinoma cell lines and xenograft mouse models, showing varied results. Some groups have demonstrated that Ad-p53 can induce apoptosis and reduce tumor volume 65-67, while another group did not observe any advantage of Ad-p53 compared to the control, revealing instead that Ad-p21 was more effective for reducing tumor volume and increasing survival 68. Most of these studies were conducted 20 years ago, and it seems that these investigators have discontinued such efforts. For the treatment of prostate cancer with gene therapy, improvements in the design of the viral vector as well as the gene transfer approach may increase efficacy, especially with respect to transduction efficiency and transgene expression.

**Figure 1:**
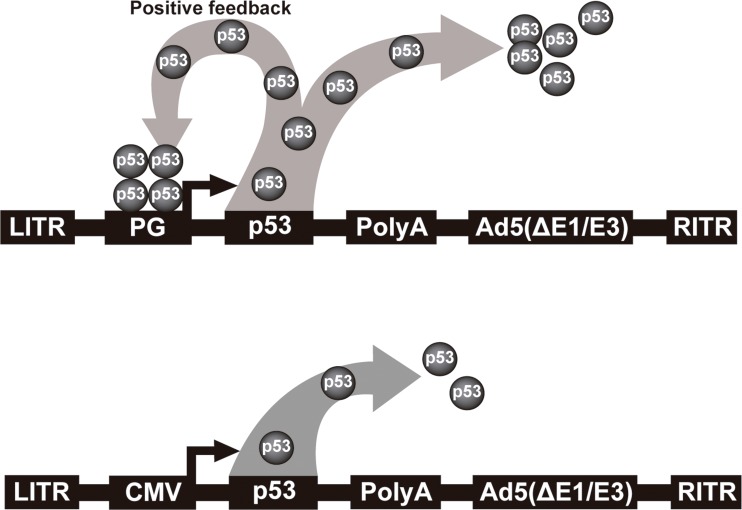
Schematic representation of non-replicating serotype 5 adenoviral vectors. Top, Ad-PGp53 featuring the p53 responsive promoter. Leaky expression of the p53 cDNA initiates binding of p53 to the PG promoter, leading to high level p53 expression due to the positive feedback mechanism. In this way, p53 serves to both drive expression as well as act as a tumor suppressor. Bottom, typical Ad-p53 vector where a constitutive promoter is used to drive expression of the p53 cDNA. LITR, left inverted terminal repeat; PG, PGTxβ chimeric p53-responsive promoter; CMV, cytomegalovirus immediate early enhancer/promoter; p53, wild-type cDNA, PolyA, polyadenylation signal; Ad5(ΔE1/E3), adenoviral genome deficient in the E1 and E3 genes; RITR, right inverted terminal repeat.

We have developed an improved Ad-p53. Instead of using a constitutive promoter, we have developed a p53-responsive promoter (PG), which was initially incorporated in a retroviral vector. This modified expression system could surpass the parental unmodified vector by up to seven-fold 69. When the p53 gene was placed under control of this PG promoter, an autoregulated positive feedback mechanism was established, leading to more robust inhibition of tumor cell proliferation 70. Next, we transferred this expression system to an adenoviral vector and observed that this promoter was 5-fold stronger than the CMV promoter 71. This vector, named Ad-PGp53, provides higher levels of p53 expression than Ad-CMVp53. A schematic representation of these two vectors is depicted in Figure 1, which are similar to the commercial vectors tested in several clinical trials. We showed that Ad-PGp53 was better able to induce cell death *in vitro* and *in vivo* than Ad-CMVp53, and *in situ* gene therapy resulted in reduced tumor volume and increased overall survival only with Ad-PGp53. In this same work, we observed that the PC3 prostate carcinoma cell line was not efficiently transduced by Ad5 72. Therefore, we made an additional improvement, incorporating the RGD motif in the fiber protein, thus creating AdRGD-PGp53, which offers both enhanced transduction efficiency in PC3 cells and a high level of p53 expression due to the positive feedback mechanism. This vector showed strong antitumor activity *in vitro* and *in vivo*, inducing high levels of reactive oxygen species (ROS), DNA damage and alteration of mitochondrial membrane permeability and resulting in apoptosis 21. Even though this improved adenoviral vector has strong antitumor activity against prostate carcinoma cell lines, even better than the versions of Ad-p53 tested in clinical trials, additional benefit may be seen if combined with chemotherapeutic agents.

### Role of p53 in the response to prostate cancer chemotherapeutic agents

For prostate cancer, the most commonly used chemotherapy agents are mitoxantrone, docetaxel and cabazitaxel. One of the first drugs used for treatment of CRPC was mitoxantrone, a synthetic anthracenedione derivative with immunomodulatory and antineoplastic activity that was approved by the FDA in 1987 for treatment of different cancers and in 1996 for prostate cancer. Clinical trials indicated that the combination of mitoxantrone plus prednisone and corticosteroid improved quality of life without affecting survival 8. Its mechanism of action involves cytotoxic activity through intercalation with DNA and inhibition of topoisomerase II 73, resulting in inhibition of replication and transcription 74,75. It also induces double-strand breaks, leading to activation of p53 and its accumulation in the nucleus 76, indicating that p53 status may be important to determine drug sensitivity. Mitoxantrone resistance can be induced by alterations in topoisomerase II or by P-glycoprotein (P-gp, MDR1, ABCB1) overexpression, which results in reduced drug accumulation inside the cell 77.

Docetaxel is the standard chemotherapeutic agent used to treat patients with prostate cancer 78. Docetaxel was synthesized from a precursor (10-deacetylbaccatin III) originally isolated from the needles of the European yew, Taxus baccata 79. It has an antimitotic effect by binding to free tubulin, promoting the formation of stable microtubules, preventing depolymerization and therefore inhibiting mitosis and inducing apoptosis 80. Collapse of microtubules results in the induction of p53, activation or inactivation of a variety of protein kinases and inhibition of cyclin-dependent kinases, resulting in cell cycle arrest in the G2/M phase. Other proapoptotic activities of docetaxel include downregulation of Bcl-2, upregulation of p53 and/or p21^WAF-1^ and induction of the phosphorylation of Bcl-X(L)/Bcl-2 members 81. Compared to wild-type p53 cells, prostate cancer cells expressing mutant p53 demonstrated reduced sensitivity in response to docetaxel, indicating that functional p53 is essential for sensitivity to docetaxel in prostate cancer cells 82,83. Docetaxel is an effective therapy against prostate cancer, but in some cases, it fails, requiring the use of alternative drugs, including cabazitaxel. Drug resistance is a major barrier for the use of docetaxel, and overcoming this impediment has been a challenge 84. The mechanisms of resistance include overexpression of P-gp 85 and altered beta-tubulin isotypes, as well as deregulation of cell survival and transcription factors 85,86.

Cabazitaxel is a taxane approved by FDA in June 2010 for treatment of prostate cancer subsequent to the use of docetaxel. Like docetaxel, cabazitaxel suppresses the dynamics of microtubules, resulting in inhibition of proliferation and cellular arrest by inducing mitotic spindle deformity. However, cabazitaxel is more efficient 87,88 since it has lower affinity for P-gp and remains inside the cell for more time 89. Resistance to cabazitaxel has been noted, though little is known about the mechanism 90. Even so, the ETS-related gene is overexpressed in prostate cells and leads to resistance to cabazitaxel treatment 91 by aborting p53 function, deregulating apoptosis, overexpressing HER2 and inhibiting tumor cell permeability 92.

### Combined therapy

The combination of Ad-p53 with chemotherapy may benefit both approaches. In a phase II clinical trial, NSCLC patients were treated with Ad-p53, docetaxel or a combination of both, and the median survival time was 7.7 months for patients who received both therapies and 5.9 months for patients who received only docetaxel 93. Patients with stage III or IV oral carcinoma were treated with Ad-p53 and chemotherapy (carboplatin, bleomycin and methotrexate), and the patients with stage III disease treated with the combined therapy had increased survival 94. Clinical trials combining Ad-p53 and chemotherapy provided a synergistic effect 93-98 of reducing side effects and increasing the quality of life and disease control compared to patients treated with only chemotherapy 99. The combination of Ad-p53 with chemotherapy may provide a therapeutic advantage in prostate carcinoma.

There is an intimate relationship between chemotherapy and p53, where the cellular p53 status is important for the prediction of drug efficacy. In ovarian cancer cells, the combination of Ad-p53 and docetaxel was positive only in cells expressing mutant p53, while the treatment was ineffective in cells with wild-type p53 status 100. In NSCLC, the combination of radiotherapy, docetaxel and Ad-p53 reduced tumor growth 101,102 regardless of the cell p53 status 103. Interestingly, Ad-p53 may be especially advantageous in chemoresistant cells since breast cancer cell lines resistant to mitoxantrone were shown to be more sensitive to Ad-p53 compared to drug-sensitive cells 104.

The combination of Ad-p53 and docetaxel resulted in enhanced antitumor effects in a murine model of HNSCC 105. Docetaxel was shown to upregulate CAR in HNSCC cells and cooperate with Ad-p53 to increase the expression of bax and the cleavage of PARP and caspase-3 106. At the same time, Ad-p53 also favors chemotherapeutic activity by suppressing hepatic enzymes and reducing docetaxel clearance 107. In prostate cancer, the combination of Ad-p53 and cisplatin reduced tumor volume in a xenograft mouse model 108. *In vivo*, the combination of antisense clusterin oligodeoxynucleotides, mitoxantrone and Ad-p53 eradicated subcutaneous and orthotopic PC3 tumors 109. Docetaxel combined with CV787 (PSA+ conditionally replicating adenovirus) synergistically reduced prostate cancer in a xenograft mouse model, where the combinatorial treatment increased the expression of p53 27. In prostate cancer cell lines, docetaxel and paclitaxel were shown to increase adenoviral transgene expression 110. The combination of chemotherapeutic drugs (cisplatin, docetaxel, mitoxantrone, paclitaxel and etoposide) with an oncolytic adenovirus showed a synergistic response in prostate cancer cell lines; in particular, docetaxel and mitoxantrone were shown to increase viral uptake, exhibiting a trend of increased levels of integrin αvβ3/β5 after treatment of DU145 and LNCaP cells with either drug, as well as a trend of increased CAR expression in PC3 cells. The combination of oncolytic adenovirus and docetaxel prolonged overall survival and reduced tumor volume 111.

Prostate cancer is one of the most important tumors in men. Development of new treatments has shown promising results, including gene transfer approaches as an appealing alternative. Thus, gene therapy is slowly regaining lost territory in the treatment of prostate cancer. The use of prostate cancer-specific oncolytic viruses and suicide gene therapy has reached clinical trials. Adenoviruses expressing the tumor suppressor p53 are employed for HNSCC, but their use has been limited in prostate cancer. We have shown that improvements in the transgene expression system and alteration of viral tropism may improve the suppressor activity of an Ad-p53. Even so, gene therapy may work in cooperation with traditional chemotherapy, benefiting both approaches and bringing about synergistic activity as an effective prostate cancer treatment.

## AUTHOR CONTRIBUTIONS

Tamura RE, Luna IV, Lana MG and Strauss BE elaborated and reviewed text. Luna IV designed the figure.
